# Study of the Acidic, Basic, and Thermal Degradation Kinetics of Three Antihypertensive Drugs—Individually and in Combination

**DOI:** 10.3390/pharmaceutics16111410

**Published:** 2024-11-02

**Authors:** Nebojša Mandić-Kovacević, Irena Kasagić-Vujanović, Biljana Gatarić, Ranko Škrbić, Ana Popović Bijelić

**Affiliations:** 1 Department of Pharmacy, Faculty of Medicine, University of Banja Luka, Save Mrkalja 14, 78000 Banja Luka, The Republic of Srpska, Bosnia and Herzegovina; 2 Centre for Biomedical Research, Faculty of Medicine, University of Banja Luka, Save Mrkalja 14, 78000 Banja Luka, The Republic of Srpska, Bosnia and Herzegovina; ranko.skrbic@med.unibl.org; 3 Department of Drug Analysis, Faculty of Medicine, University of Banja Luka, Save Mrkalja 14, 78000 Banja Luka, The Republic of Srpska, Bosnia and Herzegovina; irena.kasagic.vujanovic@med.unibl.org; 4 Department of Pharmaceutical Technology and Cosmetology, Faculty of Medicine, University of Banja Luka, Save Mrkalja 14, 78000 Banja Luka, The Republic of Srpska, Bosnia and Herzegovina; biljana.gataric@med.unibl.org; 5 Department of Pharmacology and Toxicology, Faculty of Medicine, University of Banja Luka, Save Mrkalja 14, 78000 Banja Luka, The Republic of Srpska, Bosnia and Herzegovina; 6 Department of Pathologic Physiology, First Moscow State Medical University I.M. Sechenov, 119435 Moscow, Russia; 7 Academy of Sciences and Arts of the Republic of Srpska, 78000 Banja Luka, The Republic of Srpska, Bosnia and Herzegovina; 8 Faculty of Physical Chemistry, University of Belgrade, Studentski Trg 12–16, 11158 Belgrade, Serbia; ana@ffh.bg.ac.rs

**Keywords:** perindopril tert-butylamine, amlodipine besylate, indapamide, stability, kinetics, fixed drug combinations

## Abstract

Background/Objectives: The importance of fixed-dose combinations (FDCs) for the treatment of hypertension is well established. However, from a stability perspective, FDCs present a challenge since the degradation of one active pharmaceutical ingredient (API) can be affected by the presence of another API. The aim of this study was to compare the degradation behaviors and evaluate the degradation kinetics of three antihypertensive drugs, perindopril tert-butylamine (PER), amlodipine besylate (AML), and indapamide (IND). Methods: The degradation processes were studied using the previously developed reverse phase high-performance liquid chromatographic (RP-HPLC) method after exposing each drug individually, as well as the combinations of two/three drugs, to different stress factors, such as light, oxidation, acidic, basic, or neutral pH values at different temperatures. Results: The results show that PER is most unstable under basic conditions and that AML displays a negative, while IND displays a positive effect, on PER stability when combined. AML is most affected by basic conditions and oxidation, and its stability is affected by both drugs positively; IND undergoes extreme photolysis, which is positively affected by AML but negatively by PER. Conclusions: Great care must be taken when formulating FDCs with these three drugs, as well as solutions or oral suspensions adjusted for geriatric or pediatric populations, since the stability of all three drugs is greatly affected by pH conditions, as well as light or oxidation factors and their interactions.

## 1. Introduction

During the drug development process, it is essential to perform stability studies for the active pharmaceutical ingredient (API) because API will effectively exhibit its activity at the target site only if it remains in its active form. Chemical instability of API can affect its bioavailability and cause serious side effects. The International Conference on Harmonization (ICH) recommends following Q1A (R2 of ICH guidelines) for stability testing of novel drug substances and products [1]. Stability studies provide information on how the quality of a drug substance or drug product changes with time under the influence of different environmental factors such as temperature, humidity, and light. The information obtained in these studies is key in defining the safety, quality, and effectiveness of the product, as well as its shelf life [2]. Stability of drug substances is a critical step in drug development that closely aligns with the principles of Quality by Design (QbD) [3]. The process of development and formulation of a pharmaceutical product is guided by defining the quality target product profile (QTPP) and critical quality attributes (CQAs) [4]. By controlling these attributes regarding stability, i.e., physical, chemical, and biological properties, during the manufacturing process and performing risk assessment in order to understand how the changes in formulation or manufacturing process can affect CQAs, it helps to develop a robust product. Through both accelerated and real-time stability studies, design space is defined in which the stability of APIs and pharmaceutical products is maintained [5]. Since the quality of drug substances changes as a result of chemical degradation processes that are triggered by different conditions, it is important to examine the kinetic behavior of these processes. The term degradation kinetic refers to the study of the rate of drug degradation [6]. The data collected in these types of studies enables better understanding of the mechanisms involved in drug degradation. Furthermore, it helps with selecting appropriate packaging and storage conditions that improve drug shelf life [6,7]. According to the ICH guidelines recommendations for accelerated stability studies, it would take at least half a year of laboratory work to acquire preliminary stability data. During the process of preclinical development of new medicines, it is crucial to shorten the decision-making process that pharmaceutical companies undergo concerning the quality of the new drug [8]. By developing new and improved tools, such as Accelerated Predictive Stability studies (APSs) and lean stability strategies, in order to increase the rate of the degradation of APIs under different storage and formulation conditions, this entire process becomes more cost-efficient and less time-consuming. Using these tools enables the pharmaceutical industry to establish scientific and risk-based protocols with the focus on individual stability related quality attributes, which ideally include only shelf life limiting attributes. Through isoconversion and different mathematical approximations, the results can enable the extrapolation of shelf life to different conditions [9,10]. Drug degradation processes are generally described with zero-, first-, and second-order kinetics. In zero-order kinetics, the rate of a reaction is not dependent on the concentration of the reactants, i.e., the drug and the stressor [11]. The rate of first-order degradation of drugs under different environmental conditions may be measured by observing the drug’s initial concentration decrease over time [2]. In second-order kinetics, the rate of drug degradation is determined from the changes in the concentrations of the drug and the stressor. The degradation of most pharmaceutical compounds is described using first-order kinetics. Although the first order of a reaction is mostly observed in drug degradation, in reality, they are second-order processes. In these reactions, one reactant is in large excess, which is why a change in the concentration of that reactant is insignificant and presumed to be first-order of reaction [11].

**Figure 1:**
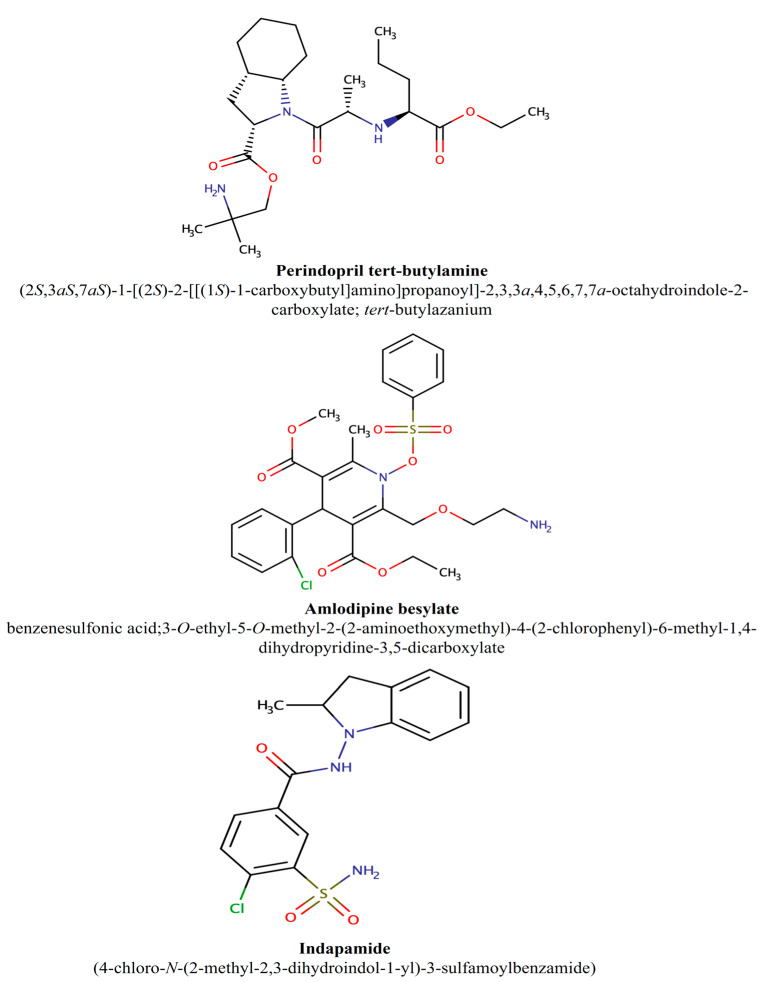
Chemical structures and IUPAC names of substances investigated in this work.

Multiple drugs often need to be used in order to achieve adequate blood pressure control for most patients [12]. Several clinical guidelines for hypertension, including the guidelines from Europe, the United Kingdom, the United States, Canada, and Latin America, have recognized the use of more than one drug in the initial management of hypertension under certain instances [13,14,15,16,17]. These guidelines recommend starting with two drugs, either as separate agents or with an FDC, for patients with blood pressure over 160/100 mm Hg or whose blood pressure is more than 20/10 mm Hg higher than the targeted value [13,14,17,18]. There is increasing interest for introducing combination drug therapy far earlier in the treatment of hypertension, and the importance of fixed-dose combinations (FDCs) for the treatment of hypertension is recognized worldwide by leading medical authorities [14,19]. This approach has been shown to improve medication tolerability, adherence, and blood pressure control and is associated with lower pill burden and reduced health care costs [20]. In addition, current guidelines in Canada recommend changing multiple-pill combinations with FDCs in the treatment of hypertension [15]. One additional benefit of using complementary classes of antihypertensive drugs as FDCs is the removal of ethnicity, race, and age in therapy algorithms [21]. However, FDCs present a challenge from the stability perspective since the degradation of one active ingredient may not only be accelerated in the presence of another API, but it may also change its degradation pathway and lead to the generation of a new degradation product. Therefore, the aim of this study was to compare the degradation behavior and evaluate the degradation processes from a kinetic perspective of three antihypertensive drugs: perindopril tert-butylamine, indapamide, and amlodipine besylate (Figure 1), individually and in combination, all in line with the approach of APSs and lean stability. The degradation processes were studied using the previously developed RP-HPLC method [22] after subjecting each drug individually or the two or three drug combinations to different stress factors, including pH, light, and oxidation.

Thus far, there are only a few studies reporting on the degradation kinetics of perindopril tert-butylamine [23,24,25,26], with none of them involving the FDC of the three drugs, only examining the stability of PER alone. Additionally, indapamide [27] and amlodipine besylate [28] were found to have been examined in combination with other antihypertensive or antilipemic drugs; no studies have been performed with all three drugs or their combinations in order to better understand their interactions. This study provides new insights into the stability and the degradation kinetics of their combinations, including the formulation containing all three active substances, making way for further research and potential for the use of this FDC in solving the problem of pediatric or geriatric formulations where this therapy is needed.

## 2. Materials and Methods

### 2.1. Chemicals

Pharmacopoeial standards (Ph. Eur.) of perindopril tert-butylamine, amlodipine besylate, and indapamide were purchased from Sigma-Aldrich, St. Louis, MO, USA. Acetonitrile (Fisher Scientific, Leicestershire, UK) was of HPLC grade. Potassium dihydrogen phosphate, potassium hydrogen phosphate, 85% o-phosphoric acid (*w*/*w*), 37% (*w*/*w*) hydrochloric acid, sodium hydroxide (Lachner, Neratovice, Czech Republic), and hydrogen peroxide 30% (*w*/*w*) (Sigma-Aldrich, USA) were of analytical grade. Water HPLC grade was attained using Thermo Scientific™ Barnstead™ LabTower™ EDI (Waltham, MA, USA) Water Purification System.

### 2.2. Kinetic Studies

#### 2.2.1. Sample Preparation for Degradation Kinetic Studies

Stock solutions of perindopril tert-butylamine, amlodipine besylate, and indapamide were prepared in the 50/50 (*v*/*v*) acetonitrile/HPLC water mixture in concentrations of 1 mg mL^−1^ and were used for the kinetic studies. Working solutions of all three drugs individually and in binary or tertiary mixtures were prepared by diluting 1 mL of the stock solution with a selected stress reagent (sodium hydroxide solution, hydrochloric acid solution, ultra-pure water, hydrogen peroxide) to make a final 100 µg mL^−1^ concentration of each drug. The influence of light on drug(s) degradation was performed following the ICH guideline [29], with the working solution(s) made up to the concentration of 100 µg mL^−1^ of each drug using mobile phase; the influence of temperature on the drug(s) degradation profile was evaluated by exposing 100 µg mL^−1^ drug solution(s) to different temperatures using heating chambers.

#### 2.2.2. Drug Stability Testing

All samples were kept at room temperature and in the dark, except for the photolytic degradation testing, and were sampled at selected time intervals (0, 30, and 60 min; 24, 48, and 72 h) and the remaining concentration was determined using the previously described RP-HPLC method [22]. Along with the working solutions, blank solutions containing the mobile phase and the stress agent were also sampled at the same time points in order to subtract the potential influence on the drug content.

Samples were subjected to basic, acidic, and neutral hydrolysis at room temperature and at 45 °C and 65 °C during 72 h, as well as photodegradation in a Memmert ICH 110L chamber equipped with fluorescent lamps with cold white light corresponding to daylight and UV radiation in the spectral range 320–400 nm for UV light exposure. The effect of oxidation on three drugs was examined using hydrogen peroxide solution as an oxidizing agent.

#### 2.2.3. Kinetic Calculations

The concentrations of all three drugs in the treated samples were determined using appropriate calibration curves, and adequate graphs were constructed in order to determine the order of the degradation reaction as well as the corresponding kinetic parameters (rate of the reaction, activation energy, free activation energy, enthalpy and entropy of activation, degradation half-time, and shelf life).

By constructing graphs for different order kinetics (for zero-order: the concentration expressed in units [mM] as a function of time expressed in units [h]; for first-order: the *ln* value of the concentration as a function of time; for second-order: the inverse value of the concentration as a function of time) and determining the highest correlation factor, the order of the reaction was determined. The degradation rate constants were determined from the slope. From these rate constants and the reaction orders, it was possible to calculate the degradation half-times and shelf life using Equations (1)–(3).

(1) $$\mathrm{zero order}\;t_{1/2}=\frac{A_{0}}{2\cdot k},\;t_{90}=\frac{A_{0}}{10\cdot k}$$

(2) $$\mathrm{first order}\;t_{1/2}=\frac{ln2}{k},\;t_{90}=\frac{0.105}{k}$$

(3) $$\mathrm{second order}\;t_{1/2}=\frac{1}{k\cdot A_{0}},\;t_{90}=\frac{0.111}{k\cdot A_{0}}$$

where *A*_0_ is the concentration of the drug at the beginning of the experiment and *k* is the degradation rate constant [11,30,31].

Using the Arrhenius Equation (4):

(4) $$k=A\cdot e^{\frac{-Ea}{RT}}$$

where *E_a_* is the Arrhenius activation energy, *R* is the universal gas constant 8.314 J mol^−1^K^−1^, *T* is temperature in K and *A* is the pre-exponential factor [30]; and the Eyring Equation (5) [8,27,28]:

(5) $$k=\frac{k_{B}\cdot T}{h}\cdot e^{\frac{-\Delta G^{‡}}{RT}}$$

where *k_B_* is the Boltzmann constant (1.380649 × 10^−23^ J K^−1^), *h* is Planck constant (6.62607015 × 10^−34^ J s) and *ΔG^‡^* is the free energy of activation, defined by Equations (6) and (7) [11,30,31]:

(6) $${\Delta G}^{‡}={\Delta H}^{‡}-T\Delta S$$

(7) $${\Delta H}^{‡}=E_{a}-RT$$

### 2.3. HPLC Method

**Figure 2:**
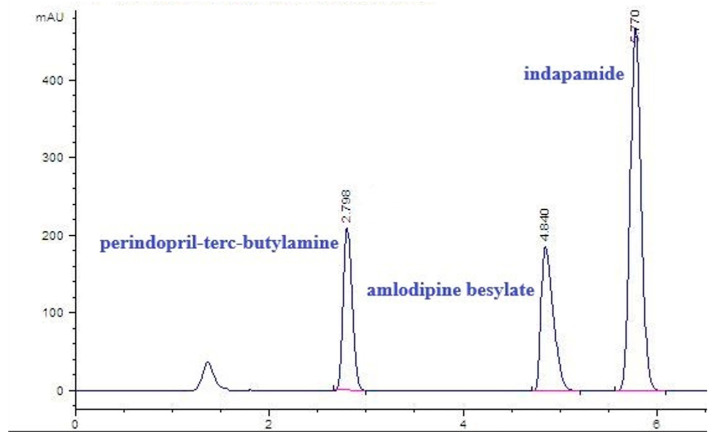
Chromatogram of the three drugs perindopril tert-butylamine (PER), amlodipine besylate (AML), and indapamide (IND).

The samples were analyzed with the previously developed RP-HPLC method [22]. Briefly, the analyses were performed on ZORBAX Eclipse XDB-C18 column (150 mm × 4.6 cm, 5 µm particle size), the mobile phase comprising acetonitrile and phosphate buffer (30 mM, pH 2.7) in the ratio 34:66 (*v*/*v*), the flow rate of 1 mL min^−1^, injection volume of 10 µL, and UV detection at 210 nm (Figure 2).

## 3. Results and Discussion

### 3.1. Drug Stability

The aim of the first part of this study was to determine the stabilities of each of the three drugs, PER, AML, and IND, as well as the stabilities of their binary and tertiary mixtures, in the absence and presence of different stress agents.

The first step was the determination of the strength (concentration) of the stress agent that would be appropriate for the drug stability testing. For basic hydrolysis, three concentrations of NaOH were tested: 0.1, 0.01, and 0.001 M. The concentration of 0.001 M (pH 11.01) was selected as appropriate with regard to PER since it had degraded in more concentrated solutions over 90% after 72 h. The acidic hydrolysis was carried out in 1 M HCl solution (pH 0.52) because lower concentrations, 0.1 M and 0.001 M, failed to induce any significant degradation with regard to PER and IND during the 72 h period. The neutral hydrolysis was monitored in HPLC-grade water (pH 6.98), and oxidation was monitored in 3, 15, and 30% hydrogen peroxide solutions. During preliminary studies, different drug ratios were checked in different conditions in order to determine whether the degradation or impurity profiles are affected. It was concluded that since no significant changes were noticed, the drug ratio of PER:AML:IND = 1:1:1 in the arbitrary concentration of 100 µg/mL can be chosen for all experiments.

Considering that pure active pharmaceutical ingredients (APIs) can be unstable in a solid state, their chemical instability may be even more pronounced in formulations because they are more susceptible to hydrolysis when in their soluble form [32,33]. Therefore, great care must be taken when dealing with such compounds, especially concerning the pH value of the formulation environment. If one considers that many excipients are prone to being hygroscopic and even chemically reactive, thus facilitating hydrolysis or other degradation reactions, the importance of stability studies for APIs in a wide range of pH is most crucial. The specific physical properties of the drug that affect its degradation rate are mainly the lipohilicity and the pKa value, which consequently determine the drug’s solubility in water [8,34]. Generally, increased solubility leads to drugs being more susceptible to degradation in water. The log P values of PER, AML, and IND are 0.63, 0.65, and 2.60, respectively, showing that IND is lipophilic and least soluble in water, indicating that it is the most resistant of the three drugs investigated in this study to hydrolysis reactions. The pKa values of the drugs (3.79, 8.60, and 8.85, for PER, AML, and IND, respectively) dictate whether they will be in their ionized forms at specific pH values, which in turn will affect their solubility. Therefore, AML as a weak base is more susceptible to hydrolysis at low pH, being predominantly in its ionized form. PER is expected to show greater stability in acidic settings but is undergoing significant degradation at basic pH.

When considering the chemical structure of the three compounds, it can be noticed that both PER and AML as esters and IND as amide are more susceptible to basic hydrolysis than acidic, which was shown for all drugs, since they exhibited greater instability to the basic environment than to acidic. While IND as an amide is more stable in the acidic environment, AML as an ester was the most susceptible to acidic hydrolysis. AML having a dihydropyridine ring in its structure, which is highly sensitive to oxidation into pyridine, showed the highest degree of degradation by the oxidation reaction.

Under basic conditions, after the 72 h period, AML degraded the most (~36%), whereas the percent of degradation for PER and IND was ~12% and ~2%, respectively (Table 1). Additionally, IND displayed a positive influence on PER and AML stabilities in their binary mixtures. Furthermore, AML showed a positive influence on both PER and IND, while PER had a positive influence on AML and IND. The stabilities of all three drugs were ameliorated in the tertiary mixture. Therefore, the shelf life of all three drugs is expected to be prolonged when they are combined in binary and tertiary mixtures. This is most dominantly observed for PER and AML, as demonstrated by having their shelf lives quadrupled or doubled, allowing for the use in extemporaneous preparations for specific populations and adjusted doses. The degradation in basic conditions was determined to follow the second-order kinetics for all three drugs (the rate constants, degradation half-times, and shelf lives are given in Table 1).

In acidic conditions, AML was the most sensitive drug, showing ~27% degradation, in contrast to PER and IND, which had degraded only ~1% (Table 2). The influence of the drugs on each other’s stability in acidic conditions was negative in all cases, resulting in higher degradation percentages of all three drugs in their binary and tertiary mixtures. The shelf life of AML was shorter when combined with other two drugs in acidic conditions, already after 24 h, leading to the conclusion that these drug combinations need to be prepared immediately prior to their administration. The shelf life of PER and IND was significantly reduced in binary and tertiary mixtures, although their stability was maintained for at least 40–48 h. As in the case of the degradation in basic conditions, second-order kinetics were observed also in the acidic medium.

The neutral hydrolysis, as expected, caused the lowest extent of degradation of all three drugs, specifically 0.72, 1.91, and 0.25%, for PER, AML, and IND, respectively. The observed negative effects were of AML on PER stability, of PER and IND on AML stability, and of PER and AML on IND stability. Regarding the shelf life, although the drug stabilities were reduced in mixtures, t_90_ was not less than 48 h at neutral pH, thus allowing for a two-day advance preparation. While the degradation of PER and IND showed second-order kinetics, the degradation of AML followed zero-order kinetics in the neutral environment (Table 3).

#### 3.1.1. Oxidation-Induced Drug Degradation

The kinetic parameters of drug degradation due to oxidation by a 3% hydrogen peroxide solution are shown in Table 4. Higher concentrations of hydrogen peroxide, 15 and 30%, were also investigated, but were shown to be extremely powerful degrading agents. For example, the 15% solution resulted in >75% degradation of AML after 24 h. The most sensitive drug to oxidation was AML, followed by PER, and finally IND, being the most stable. After 72 h, ~23% of AML was degraded in contrast to only ~3% of PER and ~2% of IND. Both AML and IND had a negative effect on PER, whereas PER and IND had a positive effect on AML stability in the binary and in tertiary mixtures. Furthermore, PER had a negative effect on IND stability, and AML had a positive effect. The shelf life of all three drugs individually or in mixtures was not critically affected by oxidation, allowing for at least a 24 h period of stability. The oxidation-induced degradation of AML and IND was calculated to follow second-order kinetics, while for PER, zero-order kinetics were found (Table 4).

#### 3.1.2. Light-Induced Drug Degradation

The light-induced instability was most prominently displayed for IND, which had degraded >25% after 72 h, contrary to PER and AML, which had degraded only 0.58% and 0.50%, respectively. The photostability of PER was decreased in the presence of both AML and IND; the same effect was observed for AML, whereas the photostability of IND was unaltered in the presence of PER and ameliorated in the presence of AML in binary and ternary mixtures with PER. Despite the fact that t_90_ was lowered for all three drugs when in binary and tertiary mixtures, a minimum of a 24 h period of stability remained. Moreover, the shelf life of IND was prolonged when combined with AML or in the tertiary mixture, leading to the conclusion that IND should be used in combinations rather than alone when no possibility of light protection during preparation is achievable. All three drugs showed zero-order kinetics for photodegradation (Table 5).

### 3.2. Kinetic and Thermodynamic Parameters Determination

In order to calculate the kinetic and thermodynamic parameters needed for the interpretation of the degradation profile of the three drugs, their solutions in basic, acidic, and neutral pH were incubated at high temperatures (45 and 65 °C) for 72 h. As expected, higher degradation was observed at higher temperatures at all investigated pH values. The thermodynamic and kinetic parameters, E_a_, ΔH^‡^, ΔG^‡^, ΔS^‡^, k, n, t_1/2_, and t_90_ were calculated using Equations (1)–(7) (for basic conditions, Table 6; for acidic conditions, Table 7; and neutral conditions, Table 8).

The treatment of all three drugs, individually and in their binary and tertiary combinations, with the 0.001 M NaOH solution caused significant degradation during 72 h (Table 1). IND was shown to be the most stable under basic hydrolysis with only ~2% degradation after 72 h, whereas PER and AML degraded ~12% and ~36%, respectively, suggesting that they undergo basic hydrolysis [27,35,36]. For AML, the calculated *E_a_* values were the lowest among the investigated drugs in basic conditions (Table 6), and moreover, ΔS^‡^ showed the highest negative values and short degradation half-times and shelf life, all implying that AML is the most unstable drug at high pH values. It should be, however, pointed out here, that the degradation half-time for PER at 65 °C was the shortest among the three investigated drugs, suggesting that PER is extremely sensitive to higher temperatures. When combined in binary and tertiary mixtures, all three drugs showed better stability. Namely, their respective degradation percentages were lower, and degradation half-times and shelf lives were longer, possibly implying that these drugs, at basic pH values, should be used in combination rather than individually in order to maintain longer stability in pharmaceutical preparations.

The degradation under acidic conditions (1 M HCl) was most prominently exhibited by AML, alone and in the mixtures with PER and IND (Table 2). Specifically, after 72 h, 27.32% of AML was degraded, whereas only 0.93% of PER and 0.82% of IND. The stability of all three drugs was lower in binary and tertiary mixtures. The corresponding *E_a_* values were the lowest for AML, and its degradation half-time and shelf life were the shortest. PER had the most negative effect on IND stability at acidic pH, by doubling ΔS^‡^ negative values for IND when combined with PER and decreasing the *E_a_* value ~5-fold (Table 7). These results indicate that great care should be taken when combining the three drugs under acidic conditions.

The neutral hydrolysis at higher temperatures led to a decrease in the stability of all three drugs and a change in the degradation kinetics. Namely, at higher temperatures, the stability of AML was improved when in combination with PER and IND. Also, the stability of IND was better in the tertiary mixture and in the binary mixture with AML. For AML, the *E_a_* value was lower when combined with PER, and its degradation half-time and shelf life were reduced. Contrary to this, when AML was in the binary mixture with IND or in the tertiary mixture, both the *E_a_* value and the degradation half-time were increased (Table 8).

## 4. Conclusions

This study presents new insights into the stability and the degradation kinetics of perindopril tert-butylamine, amlodipine besylate, and indapamide combinations. In the first part of the study, the stabilities of each of the three drugs, PER, AML, and IND, as well as the stabilities of their binary and tertiary mixtures, were determined in the absence and presence of different stress agents. The results showed AML to be the most sensitive drug to basic, acidic, and neutral hydrolysis, as well as oxidation, while the light-induced instability was most prominently displayed for IND. When combined in binary and tertiary mixtures, the stability of all three drugs was altered under all experimental conditions. The only exception was IND, whose photostability was unaltered in the presence of PER. In the second part of the study, the solutions of the three drugs in basic, acidic, and neutral pH were incubated at high temperatures (45 and 65 °C) in order to calculate the kinetic and thermodynamic parameters needed for the interpretation of their degradation profiles. When combined in binary and tertiary mixtures, all three drugs showed better stability under basic conditions. Namely, their respective degradation percentages were lower, and degradation half-times and shelf lives were longer. On the other hand, the stability of all three drugs was lower in binary and tertiary mixtures under acidic conditions. These findings possibly imply that these drugs, at basic pH values, should be used in combination rather than individually in order to maintain longer stability in pharmaceutical preparations, while great care should be taken when combining the three drugs under acidic conditions. The findings from this study indicate that further investigations of the compatibilities and stabilities of these three drugs in combinations with other commercially available excipients and/or vehicles would likely be of great practical importance.

## Figures and Tables

**Table 1:** The percentage of degradation and the corresponding kinetic parameters (reaction order (n), rate constant (k), degradation half-time (t_1/2_) and shelf life (t_90_)) for PER, AML, and IND (individually, and in the binary and tertiary mixtures) under basic conditions (0.001 M NaOH).

	PERINDOPRIL TERT-BUTYLAMINE *			
**Time of** **degradation (h)**	**PER**	**PER + AML**	**PER + IND**	**PER + AML + IND**
0	0	0	0	0
0.5	0.12 ± 0.01	0.07 ± 0.02	0.02 ± 0.01	0.22 ± 0.10
1	1.04 ± 0.01	0.22 ± 0.07	0.06 ± 0.03	0.26 ± 0.07
24	5.17 ± 0.02	2.96 ± 0.01	1.15 ± 0.14	1.44 ± 0.08
48	8.93 ± 0.02	6.89 ± 0.11	3.46 ± 0.09	2.53 ± 0.13
72	11.37 ± 0.08	10.41 ± 0.09	4.58 ± 0.08	4.03 ± 0.05
n	II	II	II	II
k (mM^−1^h^−1^)	0.0078	0.0065	0.0027	0.0023
t_1/2_ (h)	561.91	630.44	1460.85	1803.46
t_90_ (h)	62.37	69.98	162.15	200.18
	AMLODIPINE BESYLATE *			
**Time of** **degradation (h)**	**AML**	**AML + PER**	**AML + IND**	**AML + PER + IND**
0	0	0	0	0
0.5	1.69 ±0.07	0.76 ± 0.05	0.65 ± 0.11	0.83 ± 0.05
1	3.27 ± 0.03	1.49 ± 0.06	2.42 ± 0.14	1.64 ± 0.05
24	17.36 ± 0.64	12.37 ± 0.65	11.29 ± 0.15	9.50 ± 0.51
48	27.60 ± 0.51	19.58 ± 0.43	19.59 ± 0.50	14.69 ± 0.35
72	35.72 ± 0.18	26.94 ± 0.95	26.82 ± 0.39	20.53 ± 0.66
n	II	II	II	II
k (mM^−1^h^−1^)	0.043	0.0286	0.0275	0.019
t_1/2_ (h)	131.89	198.87	200.94	282.25
t_90_ (h)	14.64	22.07	22.30	31.33
	INDAPAMIDE *			
**Time of** **degradation (h)**	**IND**	**IND + PER**	**IND + AML**	**IND + PER + AML**
0	0	0	0	0
0.5	0.28 ± 0.05	0.16 ± 0.05	0.03 ± 0.01	0.46 ± 0.09
1	0.32 ± 0.03	0.24 ± 0.04	0.14 ± 0.08	0.60 ± 0.04
24	0.85 ± 0.07	0.93 ± 0.16	0.26 ± 0.04	1.21 ± 0.12
48	1.52 ± 0.03	1.28 ± 0.04	1.05 ± 0.24	1.51 ± 0.05
72	2.34 ± 0.20	1.70 ± 0.16	1.43 ± 0.14	1.53 ± 0.03
n	II	II	II	II
k (mM^−1^h^−1^)	0.0012	0.0009	0.0008	0.0008
t_1/2_ (h)	3243.23	4343.95	4879.55	4854.71
t_90_ (h)	92.50	482.18	541.63	538.88

* The percentage of degradation is given as mean ± standard deviation of measurements performed in triplicate.

**Table 2:** The percentage of degradation and the corresponding kinetic parameters (reaction order (n), rate constant (k), degradation half-time (t_1/2_) and shelf life (t_90_)) for PER, AML, and IND (individually, and in the binary and tertiary mixtures) under acidic conditions (1 M HCl).

	PERINDOPRIL TERT-BUTYLAMINE *			
**Time of** **degradation (h)**	**PER**	**PER + AML**	**PER + IND**	**PER + AML + IND**
0	0	0	0	0
0.5	0.01 ± 0.007*	0.74 ± 0.12	0.24 ± 0.08	0.46 ± 0.10
1	0.18 ± 0.06	1.17 ± 0.16	0.54 ± 0.04	1.32 ± 0.03
24	0.37 ± 0.03	1.31 ± 0.13	0.76 ± 0.23	1.86 ± 0.14
48	0.87 ± 0.07	10.80 ± 0.20	0.86 ± 0.02	3.00 ± 0.05
72	0.93 ± 0.09	14.53 ± 0.48	1.30 ± 0.15	3.16 ± 0.10
n	II	II	II	II
k (mM^−1^h^−1^)	0.0006	0.0098	0.0007	0.0019
t_1/2_ (h)	7572.10	434.38	6945.15	2394.29
t_90_ (h)	840.50	48.22	770.91	265.77
	AMLODIPINE BESYLATE *			
**Time of** **degradation (h)**	**AML**	**AML + PER**	**AML + IND**	**AML + PER + IND**
0	0	0	0	0
0.5	2.54 ± 0.16	2.80 ± 0.19	0.72 ± 0.11	2.09 ± 0.08
1	2.76 ± 0.09	3.25 ± 0.20	3.13 ± 0.03	2.96 ± 0.14
24	11.85 ± 0.30	11.93 ± 0.62	14.34 ± 0.89	31.31 ± 1.18
48	20.78 ± 0.81	23.05 ± 1.51	27.66 ± 1.11	48.35 ± 2.18
72	27.32 ± 0.97	28.75 ± 1.26	39.77 ± 1.10	57.18 ± 0.21
n	II	II	II	II
k (mM^−1^h^−1^)	0.0295	0.0301	0.0501	0.1055
t_1/2_ (h)	196.80	193.15	117.39	53.92
t_90_ (h)	21.84	20.41	13.03	5.99
	INDAPAMIDE *			
**Time of** **degradation (h)**	**IND**	**IND + PER**	**IND + AML**	**IND + PER + AML**
0	0	0	0	0
0.5	0.10 ± 0.03	0.04 ± 0.03	0.01 ± 0.005	0.09 ± 0.02
1	0.13 ± 0.04	0.12 ± 0.03	0.04 ± 0.03	0.14 ± 0.03
24	0.28 ± 0.06	0.15 ± 0.06	0.10 ± 0.03	7.19 ± 0.75
48	0.54 ± 0.03	0.18 ± 0.07	1.49 ± 0.06	12.60 ± 0.42
72	0.82 ± 0.06	0.21 ± 0.04	1.86 ± 0.08	15.48 ± 0.55
n	II	II	II	II
k (mM^−1^h^−1^)	0.0002	0.00009	0.001	0.0097
t_1/2_ (h)	17,844.12	43,047.10	3779.39	376.70
t_90_ (h)	1980.70	4778.23	419.51	41.81

* The percentage of degradation is given as mean ± standard deviation of measurements performed in triplicate.

**Table 3:** The percentage of degradation and the corresponding kinetic parameters (reaction order (n), rate constant (k), degradation half-time (t_1/2_) and shelf life (t_90_)) for PER, AML, and IND (individually, and in the binary and tertiary mixtures) under neutral conditions.

	PERINDOPRIL TERT-BUTYLAMINE *			
**Time of** **degradation (h)**	**PER**	**PER + AML**	**PER + IND**	**PER + AML + IND**
0	0	0	0.00	0
0.5	0.01 ± 0.006	0.12 ± 0.03	0.14 ± 0.05	0.28 ± 0.03
1	0.13 ± 0.05	0.15 ± 0.06	0.17 ± 0.07	0.55 ± 0.03
24	0.39 ± 0.10	0.21 ± 0.06	0.30 ± 0.05	0.82 ± 0.09
48	0.52 ± 0.12	7.41 ± 0.41	0.41 ± 0.08	1.05 ± 0.07
72	0.72 ± 0.12	7.64 ± 0.31	0.72 ± 0.03	1.13 ± 0.09
n	II	II	II	II
k (mM^−1^h^−1^)	0.0004	0.0055	0.0002	0.0004
t_1/2_ (h)	10,786.97	788.54	21,131.51	10,950.41
t_90_ (h)	1197.35	87.33	2345.60	1215.50
	AMLODIPINE BESYLATE *			
**Time of** **degradation (h)**	**AML**	**AML + PER**	**AML + IND**	**AML + PER + IND**
0	0	0	0	0
0.5	1.05 ± 0.06	0.30 ± 0.05	0.45 ± 0.13	0.74 ± 0.08
1	1.15 ± 0.07	0.36 ± 0.08	0.65 ± 0.02	1.29 ± 0.06
24	1.42 ± 0.15	0.75 ± 0.09	1.66 ± 0.11	7.67 ± 0.39
48	1.49 ± 0.21	7.17 ± 0.70	12.21 ± 0.12	11.20 ± 0.81
72	1.91 ± 0.12	9.81 ± 0.24	13.09 ± 0.36	14.68 ± 0.36
n	0	0	0	0
k (mM h^−1^)	0.00002	0.0002	0.0002	0.0003
t_1/2_ (h)	4393.31	445.02	238.62	286.11
t_90_ (h)	878.62	89.00	47.72	57.22
	INDAPAMIDE *			
**Time of** **degradation (h)**	**IND**	**IND + PER**	**IND + AML**	**IND + PER + AML**
0	0	0	0	0
0.5	0.01 ± 0.001	0.07 ± 0.01	0.03 ± 0.01	0.18 ± 0.09
1	0.06 ± 0.03	0.15 ± 0.06	0.10 ± 0.01	0.20 ± 0.05
24	0.12 ± 0.02	0.25 ± 0.02	0.12 ± 0.03	0.50 ± 0.10
48	0.16 ± 0.05	0.35 ± 0.02	2.38 ± 0.40	1.23 ± 0.70
72	0.25 ± 0.06	0.42 ± 0.01	2.51 ± 0.48	2.86 ± 0.12
n	II	II	II	0
k	0.0001 mM^−1^h^−1^	0.0002 mM^−1^h^−1^	0.0014 mM^−1^h^−1^	0.00009 mM h^−1^
t_1/2_ (h)	36,043.88	17,307.17	2550.09	1490.27
t_90_ (h)	4000.87	1921.10	283.06	298.05

* The percentage of degradation is given as mean ± standard deviation of measurements performed in triplicate.

**Table 4:** The percentage of degradation and the corresponding kinetic parameters (reaction order (n), rate constant (k), degradation half-time (t_1/2_) and shelf life (t_90_)) for PER, AML, and IND (individually, and in the binary and tertiary mixtures) caused by oxidation by 3% hydrogen peroxide solution.

	PERINDOPRIL TERT-BUTYLAMINE *			
**Time of** **degradation (h)**	**PER**	**PER + AML**	**PER + IND**	**PER + AML + IND**
0	0	0	0	0
0.5	0.15 ± 0.04	2.92 ± 0.13	0.56 ± 0.04	0.39 ± 0.01
1	0.20 ± 0.04	2.96 ± 0.19	0.59 ± 0.20	0.65 ± 0.02
24	0.74 ± 0.16	3.50 ± 0.25	1.87 ± 0.13	0.69 ± 0.06
48	1.27 ± 0.12	8.20 ± 0.30	3.23 ± 0.16	1.59 ± 0.12
72	2.86 ± 0.14	9.78 ± 008	3.87 ± 0.14	2.65 ± 0.13
n	0	0	0	0
k (mM h^−1^)	0.00008	0.0003	0.0001	0.00007
t_1/2_ (h)	1451.27	402.87	1268.95	1689.27
t_90_ (h)	290.25	80.57	253.79	337.85
	AMLODIPINE BESYLATE *			
**Time of** **degradation (h)**	**AML**	**AML + PER**	**AML + IND**	**AML + PER + IND**
0	0	0	0	0
0.5	0.40 ± 0.09	1.250.09	0.05 ± 0.03	0.05 ± 0.02
1	0.59 ± 0.01	1.60 ± 0.17	0.85 ± 0.15	0.33 ± 0.07
24	8.97 ± 0.58	7.05 ± 0.50	9.20 ± 0.52	4.80 ± 0.41
48	16.75 ± 0.27	14.04 ± 0.03	14.13 ± 0.24	10.20 ± 1.58
72	23.33 ± 2.20	19.81 ± 0.72	20.27 ± 1.20	14.72 ± 0.60
n	II	II	II	II
k (mM^−1^h^−1^)	0.0238	0.0186	0.0386	0.0137
t_1/2_ (h)	238.03	302.56	290.79	424.32
t_90_ (h)	26.42	33.58	32.28	47.10
	INDAPAMIDE *			
**Time of** **degradation (h)**	**IND**	**IND + PER**	**IND + AML**	**IND + PER + AML**
0	0	0	0	0
0.5	0	0.30 ± 0.03	0.13 ± 0.02	0.05 ± 0.02
1	0.16 ± 0.02	0.31 ± 0.08	0.18 ± 0.03	0.32 ± 0.11
24	1.05 ± 0.06	1.49 ± 0.10	0.78 ± 0.05	1.27 ± 0.09
48	1.85 ± 0.10	7.46 ± 0.11	1.27 ± 0.21	2.44 ± 0.44
72	2.38 ± 0.05	9.40 ± 0.05	1.49 ± 0.04	3.52 ± 0.49
n	II	II	II	II
k (mM^−1^h^−1^)	0.0013	0.0065	0.0008	0.0018
t_1/2_ (h)	2777.76	554.54	4748.47	2095.57
t_90_ (h)	308.33	61.55	527.08	232.61

* The percentage of degradation is given as mean ± standard deviation of measurements performed in triplicate.

**Table 5:** The percentage of degradation and the corresponding kinetic parameters (reaction order (n), rate constant (k), degradation half-time (t_1/2_) and shelf life (t_90_)) for PER, AML, and IND (individually, and in the binary and tertiary mixtures) caused by visible and UV light.

	PERINDOPRIL TERT-BUTYLAMINE *			
**Time of** **degradation (h)**	**PER**	**PER + AML**	**PER + IND**	**PER + AML + IND**
0	0	0	0	0
0.5	0.01 ± 0.005	0.14 ± 0.05	0.01 ± 0.005	0.02 ± 0.006
1	0.09 ± 0.04	0.16 ± 0.04	0.06 ± 0.03	0.11 ± 0.03
24	0.32 ± 0.08	0.23 ± 0.06	0.54 ± 0.16	0.70 ± 0.0.08
48	0.42 ± 0.08	0.32 ± 0.07	0.81 ± 0.04	1.39 ± 0.05
72	0.58 ± 0.12	0.81 ± 0.19	1.68 ±0.21	6.83 ± 0.10
n	0	0	0	0
k (mM h^−1^)	0.00002	0.00002	0.00005	0.0002
t_1/2_ (h)	6275.00	6240.07	2473.44	634.15
t_90_ (h)	1207.30	1248.01	494.69	126.83
	AMLODIPINE BESYLATE *			
**Time of** **degradation (h)**	**AML**	**AML + PER**	**AML + IND**	**AML + PER + IND**
0	0	0	0	0
0.5	0.10 ± 0.02	0.18 ± 0.03	0.02 ± 0.015	0.01 ± 0.003
1	0.12 ± 0.03	0.21 ± 0.04	0.03 ± 0.015	0.07 ± 0.02
24	0.19 ± 0.05	0.25 ± 0.08	1.54 ± 0.19	2.23 ± 0.09
48	0.42 ± 0.02	0.42 ± 0.04	3.76 ± 0.25	7.55 ± 0.40
72	0.50 ± 0.11	0.52 ± 0.05	7.90 ± 0.14	13.84 ± 0.16
n	0	0	0	0
k (mM h^−1^)	0.00001	0.000009	0.0002	0.0003
t_1/2_ (h)	8360.54	9180.00	418.46	287.62
t_90_ (h)	1672.11	1836.00	83.69	57.52
	INDAPAMIDE *			
**Time of** **degradation (h)**	**IND**	**IND + PER**	**IND + AML**	**IND + PER + AML**
0	0	0	0	0
0.5	0.07 ± 0.02	0.03 ± 0.02	0.01 ± 0.003	0.02 ± 0.01
1	0.39 ± 0.17	0.05 ± 0.03	0.10 ± 0.04	0.05 ± 0.02
24	0.50 ± 0.05	0.39 ± 0.05	1.05 ± 0.09	0.37 ± 0.20
48	7.14 ± 0.43	7.94 ± 0.39	1.69 ± 0.10	0.76 ± 0.22
72	26.18 ± 0.40	27.54 ± 0.59	4.45 ± 0.34	1.50 ± 0.15
n	0	0	0	0
k (mM h^−1^)	0.0008	0.0009	0.0001	0.00005
t_1/2_ (h)	174.79	144.10	1315.21	2717.94
t_90_ (h)	34.96	28.82	263.04	543.59

* The percentage of degradation is given as mean ± standard deviation of measurements performed in triplicate.

**Table 6:** The thermodynamic and kinetic parameters of degradation of PER, AML, and IND under basic conditions (0.001 M NaOH), obtained at 45 and 65 °C.

		**PERINDOPRIL TERT-BUTYLAMINE**			
**Thermodynamic** **parameters**		**PER**	**PER + AML**	**PER + IND**	**PER + AML + IND**
E_a_ (kJ mol^−1^)		50.33	57.88	64.56	78.16
ΔH^‡^ (kJ mol^−1^)	45 °C	47.69	55.23	61.91	75.51
	65 °C	47.52	55.07	61.75	75.35
ΔG^‡^ (kJ mol^−1^)	45 °C	91.13	92.28	87.52	94.48
	65 °C	93.87	94.62	89.14	95.68
ΔS^‡^ (kJ mol^−1^ K^−1^)	45 °C	−0.137	−0.117	−0.081	−0.060
	65 °C	−0.137	−0.117	−0.081	−0.060
**Kinetic parameters**					
k *	45 °C	0.0071	0.0046	0.0278	0.002
	65 °C	0.0219	0.0168	0.1179	0.0115
n	45 °C	I	I	II	I
	65 °C	I	I	II	I
t_1/2_ (h)	45 °C	97.61	150.65	148.69	319.5
	65 °C	31.64	41.25	34.89	55.56
t_90_ (h)	45 °C	14.79	22.83	16.50	52.50
	65 °C	4.79	6.25	3.87	9.13
		**AMLODIPINE BESYLATE**			
**Thermodynamic** **parameters**		**AML**	**AML + PER**	**AML + IND**	**AML + PER + IND**
E_a_ (kJ mol^−1^)		22.77	3.80	16.25	5.57
ΔH^‡^ (kJ mol^−1^)	45 °C	20.13	1.16	13.61	2.92
	65 °C	19.96	0.993	13.44	2.76
ΔG^‡^ (kJ mol^−1^)	45 °C	85.03	85.22	84.99	85.79
	65 °C	89.12	90.51	89.48	91.01
ΔS^‡^ (kJ mol^−1^ K^−1^)	45 °C	−0.204	−0.264	−0.224	−0.261
	65 °C	−0.205	−0.265	−0.225	−0.261
**Kinetic parameters**					
k *	45 °C	0.0713	0.0664	0.0725	0.0535
	65 °C	0.1187	0.0723	0.1043	0.0606
n	45 °C	II	II	II	II
	65 °C	II	II	II	II
t_1/2_ (h)	45 °C	80.48	85.91	77.44	105.13
	65 °C	49.44	77.12	54.82	90.97
t_90_ (h)	45 °C	8.93	9.54	8.59	11.67
	65 °C	5.49	8.56	6.08	10.10
		**INDAPAMIDE**			
**Thermodynamic** **parameters**		**IND**	**IND + PER**	**IND + AML**	**IND + PER + AML**
E_a_ (kJ mol^−1^)		44.75	65.94	54.45	17.82
ΔH^‡^ (kJ mol^−1^)	45 °C	42.11	63.30	51.83	15.17
	65 °C	41.94	63.13	51.67	15.01
ΔG^‡^ (kJ mol^−1^)	45 °C	90.20	90.12	93.79	89.17
	65 °C	93.23	91.82	96.43	93.83
ΔS^‡^ (kJ mol^−1^ K^−1^)	45 °C	−0.151	−0.084	−0.132	−0.233
	65 °C	−0.152	−0.085	−0.132	−0.233
**Kinetic parameters**					
k *	45 °C	0.0101	0.0104	0.0026	0.0149
	65 °C	0.0275	0.0455	0.0088	0.0222
n	45 °C	II	II	II	II
	65 °C	II	II	II	II
t_1/2_ (h)	45 °C	348.52	346.10	1391.95	243.73
	65 °C	130.45	78.70	409.36	160.49
t_90_ (h)	45 °C	38.69	38.42	154.51	27.05
	65 °C	14.48	8.74	45.44	17.81

* The rate constant (k) is expressed in units [h^−1^] for the first-order reaction, and [mM^−1^ h^−1^] for the second-order reaction.

**Table 7:** The thermodynamic and kinetic parameters of degradation of PER, AML, and IND under acidic conditions (1 M HCl), obtained at 45 and 65 °C.

		**PERINDOPRIL TERT-BUTYLAMINE**			
**Thermodynamic** **parameters**		**PER**	**PER + AML**	**PER + IND**	**PER + AML + IND**
E_a_ (kJ mol^−1^)		54.68	34.68	46.00	28.15
ΔH^‡^ (kJ mol^−1^)	45 °C	52.04	32.04	43.36	25.51
	65 °C	51.87	31.87	43.19	25.34
ΔG^‡^ (kJ mol^−1^)	45 °C	93.00	88.29	93.41	87.52
	65 °C	95.42	91.83	96.56	91.43
ΔS^‡^ (kJ mol^−1^ K^−1^)	45 °C	−0.129	−0.177	−0.157	−0.195
	65 °C	−0.129	−0.177	−0.158	−0.196
**Kinetic parameters**					
k *	45 °C	0.0035	0.0208	0.003	0.0278
	65 °C	0.0119	0.0452	0.0084	0.0522
n	45 °C	I	II	I	II
	65 °C	I	II	I	II
t_1/2_ (h)	45 °C	198	197.83	231.00	146.37
	65 °C	58.23	79.86	82.50	80.73
t_90_ (h)	45 °C	30.00	21.96	35.00	16.25
	65 °C	8.82	8.86	12.50	8.96
		**AMLODIPINE BESYLATE**			
**Thermodynamic** **parameters**		**AML**	**AML + PER**	**AML + IND**	**AML + PER + IND**
E_a_ (kJ mol^−1^)		28.93	41.85	66.17	68.07
ΔH^‡^ (kJ mol^−1^)	45 °C	26.29	39.21	63.53	65.42
	65 °C	26.12	39.04	63.36	65.26
ΔG^‡^ (kJ mol^−1^)	45 °C	72.79	85.20	74.08	75.01
	65 °C	75.72	88.10	74.75	75.62
ΔS^‡^ (kJ mol^−1^ K^−1^)	45 °C	−0.146	−0.145	−0.033	−0.030
	65 °C	−0.147	−0.145	−0.034	−0.031
**Kinetic parameters**					
k *	45 °C	7.3077	0.0669	4.4853	3.1626
	65 °C	13.963	0.1707	19.723	14.509
n	45 °C	II	I	II	II
	65 °C	II	I	II	II
t_1/2_ (h)	45 °C	0.75	10.36	1.26	1.78
	65 °C	0.41	4.06	0.29	0.38
t_90_ (h)	45 °C	0.08	1.57	0.14	0.20
	65 °C	0.05	0.61	0.03	0.04
		**INDAPAMIDE**			
**Thermodynamic** **parameters**		**IND**	**IND + PER**	**IND + AML**	**IND + PER + AML**
E_a_ (kJ mol^−1^)		62.24	11.92	52.75	65.82
ΔH^‡^ (kJ mol^−1^)	45 °C	59.60	9.28	50.11	63.18
	65 °C	59.43	9.11	49.94	63.01
ΔG^‡^ (kJ mol^−1^)	45 °C	92.86	89.72	92.72	89.23
	65 °C	94.95	94.79	95.40	90.87
ΔS^‡^ (kJ mol^−1^ K^−1^)	45 °C	−0.105	−0.253	−0.134	−0.082
	65 °C	−0.105	−0.253	−0.134	−0.082
**Kinetic parameters**					
k *	45 °C	0.0037	0.0121	0.0037	0.0146
	65 °C	0.0149	0.0158	0.0127	0.0637
n	45 °C.	I	I	I	II
	65 °C	I	I	I	II
t_1/2_ (h)	45 °C	187.30	57.27	177.69	237.76
	65 °C	46.51	43.86	54.57	56.19
t_90_ (h)	45 °C	28.38	8.68	26.92	26.39
	65 °C	7.05	6.65	8.27	6.24

* The rate constant (k) is expressed in units [h^−1^] for the first-order reaction, and [mM^−1^ h^−1^] for the second-order reaction.

**Table 8:** The thermodynamic and kinetic parameters of degradation of PER, AML, and IND under neutral conditions (H_2_O), obtained at 45 and 65 °C.

		**PERINDOPRIL TERT-BUTYLAMINE**			
**Thermodynamic** **parameters**		**PER**	**PER + AML**	**PER + IND**	**PER + AML + IND**
E_a_ (kJ mol^−1^)		45.92	40.17	30.97	49.09
ΔH^‡^ (kJ mol^−1^)	45 °C	43.28	37.52	28.33	46.44
	65 °C	43.11	37.36	28.16	46.28
ΔG^‡^ (kJ mol^−1^)	45 °C	92.72	93.00	96.90	98.15
	65 °C	95.83	96.50	101.22	101.40
ΔS^‡^ (kJ mol^−1^ K^−1^)	45 °C	−0.155	−0.174	−0.216	−0.163
	65 °C	−0.156	−0.175	−0.216	−0.163
**Kinetic parameters**					
k *	45 °C	0.0039	0.0035	0.0008	0.0005
	65 °C	0.0109	0.0086	0.0016	0.0015
n	45 °C	I	I	0	0
	65 °C	I	I	0	0
t_1/2_ (h)	45 °C	177.69	198.00	165.79	238.78
	65 °C	63.58	80.58	74.22	77.81
t_90_ (h)	45 °C	26.92	30.00	33.16	47.76
	65 °C	9.63	12.21	14.84	15.56
		**AMLODIPINE BESYLATE**			
**Thermodynamic** **parameters**		**AML**	**AML + PER**	**AML + IND**	**AML + PER + IND**
E_a_ (kJ mol^−1^)		57.91	34.05	76.62	71.91
ΔH^‡^ (kJ mol^−1^)	45 °C	55.27	31.41	73.98	69.27
	65 °C	55.10	31.24	73.81	69.10
ΔG^‡^ (kJ mol^−1^)	45 °C	93.50	95.43	102.68	102.40
	65 °C	95.91	99.46	104.49	104.49
ΔS^‡^ (kJ mol^−1^ K^−1^)	45 °C	−0.120	−0.201	−0.090	−0.104
	65 °C	−0.121	−0.202	−0.091	−0.105
**Kinetic parameters**					
k *	45 °C	0.0029	0.0014	0.00009	0.0001
	65 °C	0.0106	0.003	0.0005	0.0005
n	45 °C	I	I	0	0
	65 °C	I	I	0	0
t_1/2_ (h)	45 °C	238.97	495.00	982.54	855.78
	65 °C	65.38	231.00	170.54	170.16
t_90_ (h)	45 °C	36.21	75.08	196.51	171.16
	65 °C	9.91	32.84	34.11	34.03
	**INDAPAMIDE**				
**Thermodynamic** **parameters**		**IND**	**IND + PER**	**IND + AML**	**IND + PER + AML**
E_a_ (kJ mol^−1^)		35.68	71.91	54.68	102.88
ΔH^‡^ (kJ mol^−1^)	45 °C	33.03	69.27	52.04	100.24
	65 °C	32.87	69.10	51.87	100.07
ΔG^‡^ (kJ mol^−1^)	45 °C	102.68	102.40	98.15	106.66
	65 °C	107.07	104.50	101.05	107.07
ΔS^‡^ (kJ mol^−1^ K^−1^)	45 °C	−0.219	−0.104	−0.145	−0.020
	65 °C	−0.220	−0.105	−0.146	−0.021
**Kinetic parameters**					
k *	45 °C	0.00005	0.0001	0.0005	0.00002
	65 °C	0.0002	0.0005	0.0017	0.0002
n	45 °C	0	0	II	0
	65 °C	0	0	II	0
t_1/2_ (h)	45 °C	2734.72	2713.21	7004.85	6896.66
	65 °C	697.00	553.58	2152.00	687.57
t_90_ (h)	45 °C	546.94	271.32	777.54	1379.33
	65 °C	139.40	55.36	238.87	137.51

* The rate constant (k) is expressed in units [mM h^−1^] for the zero-order reaction, [h^−1^] for the first-order reaction, and [mM^−1^ h^−1^] for the second-order reaction.

## Data Availability

Data are contained within the article.
